# Expression Patterns of Serotonin Receptors 5-HT1A, 5-HT2A, and 5-HT3A during Human Fetal Lung Development

**DOI:** 10.3390/ijms24032965

**Published:** 2023-02-03

**Authors:** Jelena Nikolić, Katarina Vukojević, Violeta Šoljić, Josip Mišković, Martina Orlović Vlaho, Mirna Saraga-Babić, Natalija Filipović

**Affiliations:** 1 Laboratory for Experimental Neurocardiology, Department of Anatomy, Histology and Embryology, University of Split School of Medicine, Šoltanska 2, 21000 Split, Croatia; 2 Mediterranean Institute for Life Sciences (MedILS), Meštrovićevo Šetalište 45, 21000 Split, Croatia; 3 Laboratory for Early Human Development, Department of Anatomy, Histology and Embryology, University of Split School of Medicine, Šoltanska 2, 21000 Split, Croatia; 4 Faculty of Health Studies, University of Mostar, 88000 Mostar, Bosnia and Herzegovina; 5 Department of Anatomy, School of Medicine, University of Mostar, 88000 Mostar, Bosnia and Herzegovina

**Keywords:** lung, fetal development, serotonin, serotonin receptors

## Abstract

We analyzed the expression of the serotonin receptors 5-HT1A, 5-HT2A, and 5-HT3A at four different stages of fetal lung development from 12 to 40 weeks of gestation, divided into four groups: the pseudoglandular stage (12–16th week of development; *n* = 8), the canalicular stage (16th–26th week of development; *n* = 7), the saccular stage (26th-36th week of development; *n* = 5), and the alveolar stage (36th–40th week of development; *n* = 5). The strongest expression of all three receptor types was found in the epithelium of the proximal airways during the pseudoglandular, canalicular, and saccular stages and in a vascular wall. 5-HT1A was also strongly expressed in the smooth muscle cells of the proximal airway. Vascular smooth muscle cells and endothelium occasionally showed a strong expression of 5-HT1A and 5-HT2A. In the alveolar stage, the expression of 5-HT1A, 5-HT2A, and 5-HT3A was detected in both type I (p1) and type II (p2) pneumocytes, with a stronger expression in p2. A significant decrease in percent the 5-HT2A area and in the integrated density was observed at the alveolar stage. On the other hand, a significant decrease in the percentage area but an increase in the integrated density was observed for 5-HT3A toward the alveolar stage, suggesting that a smaller number of cells expressed 5-HT3A but that they (p1 and p2) significantly increased their 5-HT3A expression at the alveolar stage. The results presented provided us with new data on the development and function of the serotonin system in the human fetal lung and gave us insight into their possible involvement in the pathogenesis of lung pathology, particularly that characteristic of the neonatal period.

## 1. Introduction

Lung development is divided into three major temporal stages: the embryonic stage, the fetal stage, and postnatal lung development [1]. The embryonic period is a period of organ development, while the fetal period represents the functional maturation of the lung and is divided into the pseudoglandular, canalicular, and saccular stages. In the postnatal developmental period, the lung matures into the adult form [2].

The developmental stages of the lung are mainly based on morphological criteria and overlap due to non-synchronous development [1]. During the embryonic phase of human lung development (4–7 weeks), the primary left and right lung buds form from the endoderm of the foregut and branch to form the entire lobular structure of the lung [3]. The primitive foregut divides into the esophagus and trachea, and the large airways and pleura are formed [1]. In the pseudoglandular stage (5–17 weeks), the lung continues to grow via branching morphogenesis, and the bronchial tree and much of the prospective parenchyma are formed. In the canalicular stage (16–26 weeks), the lung tissue becomes highly vascularized and, from the 24th week, respiration becomes possible due to the development of terminal sacs (primordial alveoli) at the end of the respiratory bronchioles [2]. The saccular stage (24–38 weeks) is the intermediate stage where branching morphogenesis ends and alveolarization has not yet begun. During this time, many more terminal sacs (primordial alveoli) develop. This stage may be necessary because branching morphogenesis and alveolarization are completely different genetic programs that presumably do not occur in parallel [1]. Postnatal lung development consists of the stages of classical and continued alveolarization and microvascular maturation. It occurs from 36 weeks to young adulthood (21 years) [4,5].

The function of all major organ systems, including the cardiovascular, pulmonary, gastrointestinal (GI), and urogenital systems, as well as the central nervous system (CNS), is regulated by serotonin and serotonin receptors [6]. Serotonin [5-hydroxytryptamine (5-HT)] is primarily known as a neurotransmitter that is crucial for the development and function of the CNS, where it controls sleep, appetite, mood, and other important brain processes [7,8,9]. However, as a peripheral hormone, serotonin also plays important roles in a variety of other organ systems and can act as a mitogen, vasodilator, vasoconstrictor, or mediator of inflammation [7,10].

Most of the serotonin in the body circulates in the bloodstream, is transported by platelets, and is released when they are activated [7]. It is also produced extensively by the enterochromaffin cells of the gastrointestinal tract and by neurons of the enteric nervous system (ENS) and central nervous system (CNS) [8,11]. L-tryptophan, a precursor amino acid, is converted to 5-hydroxytryptophan (or 5-HTP) by the rate-limiting enzyme tryptophan hydroxylase (TPH). Through the action of the aromatic acid decarboxylase, 5-HTP is then converted to 5-hydroxytriptamine (5-HT, serotonin) [12]. Only 1 to 2% of serotonin is produced by serotonergic neurons in the brain, while 90% is secreted by enterochromaffin cells [12]. However, recent studies have shown that lung endothelial cells in human lungs also synthesize 5-HT [13].

Serotonin receptors are widely expressed in a variety of peripheral organs. Seven receptor types (5-HT1-7) are divided into subgroups based on their sequence and pharmacological similarity [14]. To date, 14 different subtypes have been identified. With the exception of the 5-HT3 receptor, which is a ligand-gated ion channel, all are G-protein-coupled receptors (GPCRs) [11].

The modulation of respiratory rhythm and pulmonary vasoconstriction are some of the pulmonary functions of serotonin [6,15]. The involvement of serotonin and its receptors in some pulmonary pathologies has also been documented in relation to genetic and/or environmental factors such as sudden infant death syndrome (SIDS), pulmonary fibrosis, or pulmonary hypertension associated with obstructive pulmonary disease, especially in relation to the use of antidepressants or appetite suppressants acting as serotonin receptor agonists or selective serotonin reuptake inhibitors (SSRIs) during pregnancy [15,16,17]. In the early 1980s, studies on sheep fetuses provided the first pharmacological evidence that serotonin has a stimulatory effect on fetal respiration [10,18].

It was found that 5-HT immunoreactive cells are present in human lungs from the eighth week of gestation [10]. However, little is known about the role of serotonin in fetal lung development and the expression and dynamics of serotonin receptors in human lungs during development. Delaney and coworkers [10] demonstrated that the 5-HT2A receptor is expressed in fetal sheep lungs and that intrapulmonary infusions of the 5-HT2A receptor antagonist ketanserin increased pulmonary blood flow in chronically instrumented fetal sheep. Later, they found that 5-HT and SSRIs further increased pulmonary vascular resistance in a sheep model of persistent pulmonary hypertension of the newborn (PPHN) and that the blockage of 5-HT2A receptors by ketanserin decreased pulmonary vascular resistance [19]. They also found that in experimental PPHN, the production of serotonin in lung endothelial cells and the expression of tryptophan hydroxylase 1 (Tph1, a key enzyme in serotonin production) and 5-HT2A receptors are increased [19]. To understand the relationship between 5-HT and respiratory disorders, it is necessary to investigate the role and distribution of 5-HT receptors. However, only one study has examined serotonin receptor expression in the human fetal lung. Hodge and colleagues examined 5-HT4 receptor expression in samples of human fetal lungs from 19 days to 19 weeks of gestation and found a trend toward an increase in 5-HT4 receptor protein expression from the embryonic to the pseudoglandular stage and a decrease at the canalicular stage [20]. Another study by Castro and coworkers examined serotonin transporter (SERT) expression in normal human fetal lungs from 12 to 40 weeks of gestation and in postnatal lungs, as well as in the lungs of neonates and children with pulmonary hypertension of different etiologies [21]. They found that the expression of SERT begins at the late saccular stage and peaks at or just before birth, suggesting a regulatory role of SERT in pulmonary vascular growth and remodeling, especially at the critical point just before birth. They pointed out that the identification of SERT/serotonin pathway defects could also guide new therapeutic strategies targeting their functions in infants with pulmonary hypertension and its lethal form—alveolar capillary dysplasia with pulmonary vein malposition.

In this sense, knowledge of the dynamics of the expression of different types of serotonin receptors during the normal fetal development of the human lung would be a prerequisite for determining abnormalities in the serotonin pathway that may impair development and lead to respiratory disorders in early postnatal life. Therefore, we aimed to study the expression of three types of serotonin receptors (5-HT1A, 5-HT2A, and 5-HT3A) in the human lung at different stages of development. We believe that knowledge of the spatiotemporal distribution of 5-HT3A receptors in the fetal lung during different stages of development will allow us to better understand the role of serotonin in lung development, its relationship to neonatal lung pathology, and potential therapeutic approaches.

## 2. Results

### 2.1. General Remarks

**Figure 1:**
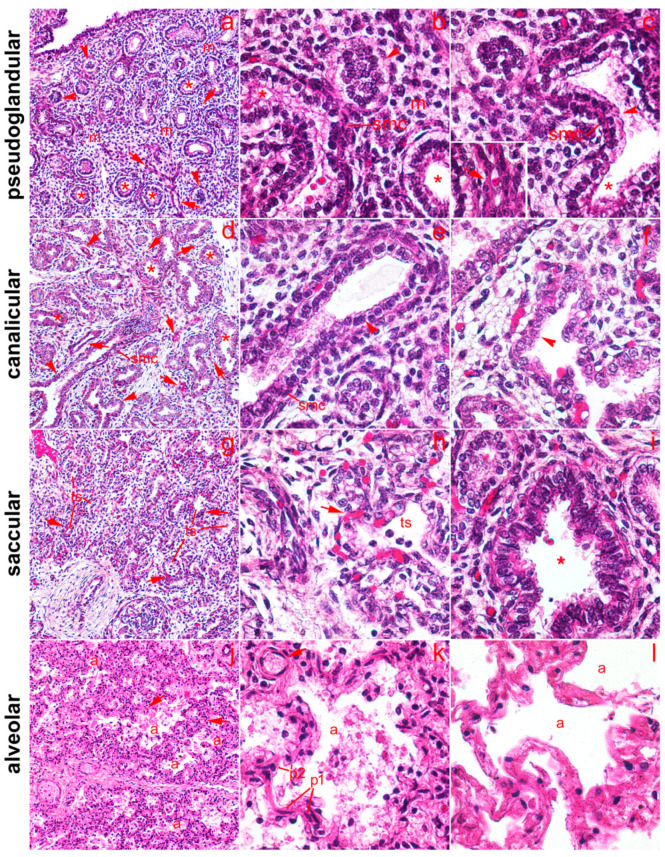
Fetal lungs at various stages of development. (**a**–**c**) Pseudoglandular stage. (**a**) 100× magnification shows typical appearance of lung in pseudoglandular stage with small lumina of proximal airways (asterisks) and abundant mesenchyme (m) with some blood capillaries (arrows). Lumina of some proximal airways are not yet open (arrowheads). (**b**,**c**) Details of lung histology in the pseudoglandular phase (400× magnification): proximal airways with columnar epithelium (asterisks); transition from proximal to distal airways (arrowheads), shown in (**c**), where the transition from columnar to cuboidal epithelium is clearly visible; m—mesenchyme; smc—smooth muscle cells visible beneath the proximal airway epithelium. Inset in (**c**)—detail of capillary (arrow) with red blood cells. (**d**–**f**) Canalicular stage. Larger lumina of proximal airway and increased vascularity can be seen. (**d**) 100× magnification; (**e**,**f**) 400× magnification. (**f**) The epithelium lining the distal airways is cuboidal (arrowhead). (**g**–**i**) Saccular stage (terminal sac), showing the development of terminal sacs (ts) with differentiation of type I and II pneumocytes from the cuboidal epithelium and the invasion of capillaries (arrows) are clearly visible. (**g**) 100× magnification; (**h**,**i**) 400× magnification. (**h**) Detail of end sac showing differentiation of type I and II pneumocytes from cuboidal epithelium; (**i**) detail of proximal airway showing columnar epithelium. (**j**,**k**,**l**) Alveolar stage, showing development of characteristic alveoli, shown in (**a**), with thin septal walls and differentiation of type I (p1) and II pneumocytes (p2). (**j**) 100× magnification; (**k**,**l**) 400× magnification. (**l**) Details of developed alveoli (**a**).

We analyzed the expression of the serotonin receptors 5-HT1A, 5-HT2A, and 5-HT3A at four different stages of fetal lung development from 12 to 40 weeks of gestation. Morphological differences between the stages were determined by hematoxylin-eosin staining (Figure 1). The histological sections of the fetal lungs examined showed typical features for the different stages of human fetal lung development (Figure 1). The pseudoglandular stage showed the typical appearance of the lung with small lumina of the proximal airways and an abundant mesenchyme with some blood capillaries. The lumina of some airways were not yet open. In the pseudoglandular stage, the proximal airways could be seen with a columnar epithelium, developing terminal bronchioles with the transition from a columnar to cuboidal epithelium and smooth muscle cells under the epithelium of the proximal airways. In the canalicular stage, the lungs could be seen with larger lumina of the proximal airways and an increased vascularity. The epithelium lining the distal airways was cuboidal. In the saccular (terminal sac) stage, the development of terminal sacs was seen with a differentiation of type I and II pneumocytes from the cuboidal epithelium and infiltration of capillaries, whereas in the alveolar stage, the development of characteristic alveoli with thin septal walls and a differentiation of type I and II pneumocytes was seen.

### 2.2. 5-HT1A Expression

**Figure 2:**
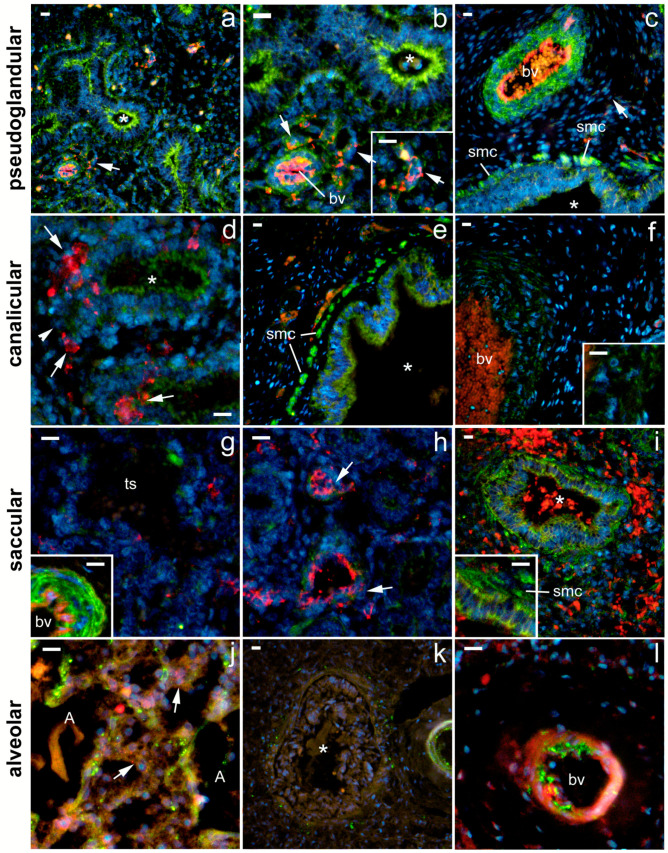
Immunofluorescence staining of fetal human lungs at different developmental stages: pseudoglandular (**a**–**c**), canalicular (**d**–**f**), saccular (**g**–**i**), and alveolar (**j**–**l**) with 5-HT1A and CD31/PECAM markers. Nuclear staining DNA DAPI is shown in parallel with 5-HT1A and CD31/PECAM immunofluorescence (merge). Green staining—positive 5-HT1A receptor expression, red staining—CD31/PECAM-1 expression, and blue staining—nuclei stained with 6-diamidino-2-phenylindole dihydrochloride (DAPI). Images were taken at 400× magnification; (**a**,**c**,**e**,**f**,**i**,**k**) downsized images. Scale bar = 20 µm. Asterisks—proximal airways/bronchioles; arrows—blood capillaries; arrowheads—distal airway bronchioles, cuboidal epithelium lining the air spaces; bv—blood vessel (mostly artery); smc—smooth muscle cells; ts—terminal sacs; A—alveoli.

**Figure 3:**
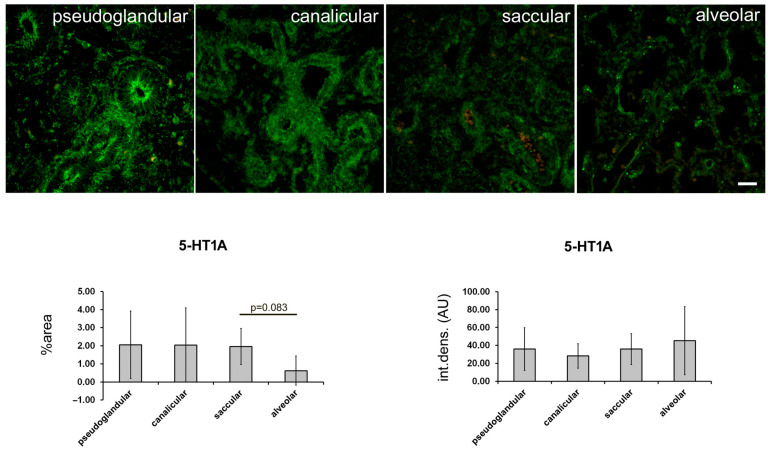
Quantification of 5-HT1A expression at different stages of fetal lung development. Immunofluorescence staining with 5-HT1A antibodies was performed. Representative images for different stages of lung development are shown. Green fluorescent staining—positive 5-HT1A receptor expression. Images were acquired at 400× magnification. Scale bar = 50 µm. The percentage of a sectional area with positive signal (% area) and the integrated density (int. dens.; arbitrary fluorescence units—AU) were measured and are presented in graphs.

During the pseudoglandular developmental stage, the strongest 5-HTA1 expression was found in the smooth muscle cells of the proximal airways and the arterial tunica media (Figure 2). In addition, strong 5-HT1A expression was observed in the epithelium of the proximal airways and in the endothelium of the developing arteries, where it was more pronounced compared with the capillary endothelium (Figure 2; Table 1). The strong expression of 5-HT1A in the proximal airways was maintained during the canalicular and saccular stages but disappeared from the bronchioles during the alveolar stage (Figure 2; Table 1). However, its expression in the smooth muscle cells of the proximal airways was strong during the pseudoglandular, canalicular, and saccular stages and remained at a moderate level during the alveolar stage of development. On the other hand, 5-HTA1 expression in the vascular wall was strongest in the muscle layer during the pseudoglandular, saccular, and alveolar stages, whereas it transiently decreased during the canalicular stage (Figure 2, Table 1). Arterial endothelial expression was strongest in the pseudoglandular and alveolar stages. Endothelial expression in capillaries changed from moderate in the pseudoglandular stage to low in all the subsequent developmental stages. Moderate-to-low 5-HTA1 expression was also seen in the developing airspaces (in the canalicular phase) and in the terminal sacs (in the saccular phase). Because of the high variability of expression in the different structures, no significant percentage difference was detected among the signal or signal intensity of 5-HTA1 (measured as integrated density) (Figure 3).

### 2.3. 5-HT2A Expression

**Figure 4:**
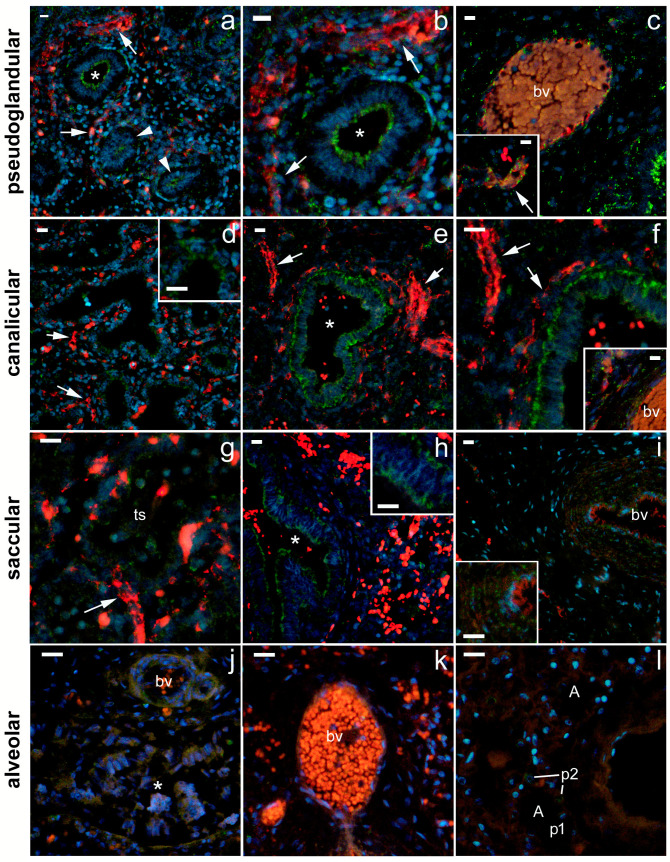
Immunofluorescence staining of fetal human lungs in different developmental stages: pseudoglandular (**a**–**c**), canalicular (**d**–**f**), saccular (**g**–**i**), and alveolar (**j**–**l**) with 5-HT1A and CD31/PECAM markers. Nuclear DNA 6-diamidino-2-phenylindole dihydrochloride (DAPI) staining (blue) merged with 5-HT2A receptor (green) and CD31/PECAM (red) immunofluorescence is shown in parallel (merge). Pictures were captured at 400× magnification; (**a**,**c**,**d**) (main figure), (**e**,**h**) (main figure), and (**i**) (main figure)— downsized. Scale bars = 20 µm. Asterisk—proximal airways/bronchioli; arrows—blood capillaries; arrowheads—distal airways, cuboidal epithelium lining the airspaces; bv—blood vessel (mostly artery); smc—smooth muscle cells; ts—terminal sacs; A—alveolae.

**Figure 5:**
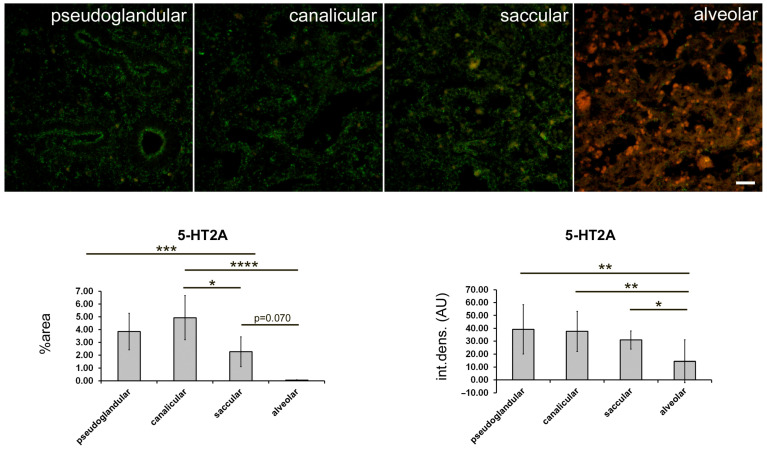
Quantification of 5-HT2A expression at different stages of fetal lung development. Immunofluorescence with 5-HT1A antibodies was performed. Representative images for different stages of lung development are shown. Green fluorescent staining—positive 5-HT2A receptor expression. Images were acquired at 400× magnification. Scale bar = 50 µm. Percentage of a sectional area with positive signal (% area) and integrated density (int. dens.; arbitrary fluorescence units—AU) were measured and are shown in graphs. * *p* < 0.05; ** *p* < 0.01; *** *p* < 0.001; **** *p* < 0.0001 between indicated developmental stages (bars).

The strongest expression of 5-HTA2 was found in the canalicular stage, particularly in the proximal airway epithelium and arterial endothelium and in the smooth muscle wall (Figure 4, Table 1). The expression of 5-HTA2 in the proximal airway epithelium was moderate at the pseudoglandular and saccular stages and almost disappeared by the time the bronchioles developed at the alveolar stage (Figure 4; Table 1). Moderate 5-HTA1 expression was observed in the SMC of the proximal airways and in the capillary endothelium during the pseudoglandular stage and in the SMC of the arterial wall during the saccular stage. In addition, low-to-moderate 5-HTA2 expression was also present in the developing distal airways (in the canalicular stage) and end sacs (in the saccular stage). However, at the alveolar stage, 5-HTA2 expression completely disappeared from the vascular wall and smooth muscle cells of the bronchioles (Figure 4; Table 1). A statistically significant decrease in the percentage area under the signal was observed in the saccular (compared with the canalicular) stage (*p* < 0.05) and also in the alveolar stage, in comparison with pseudoglandular (*p* < 0.001) and canalicular stage (*p* < 0.0001; Figure 5). The integrated density (a measure of the intensity of the signal) was significantly lower at the alveolar stage compared with all the other developmental stages (*p* < 0.05 to *p* < 0.01; Figure 5).

### 2.4. 5-HT3A Expression

**Figure 6:**
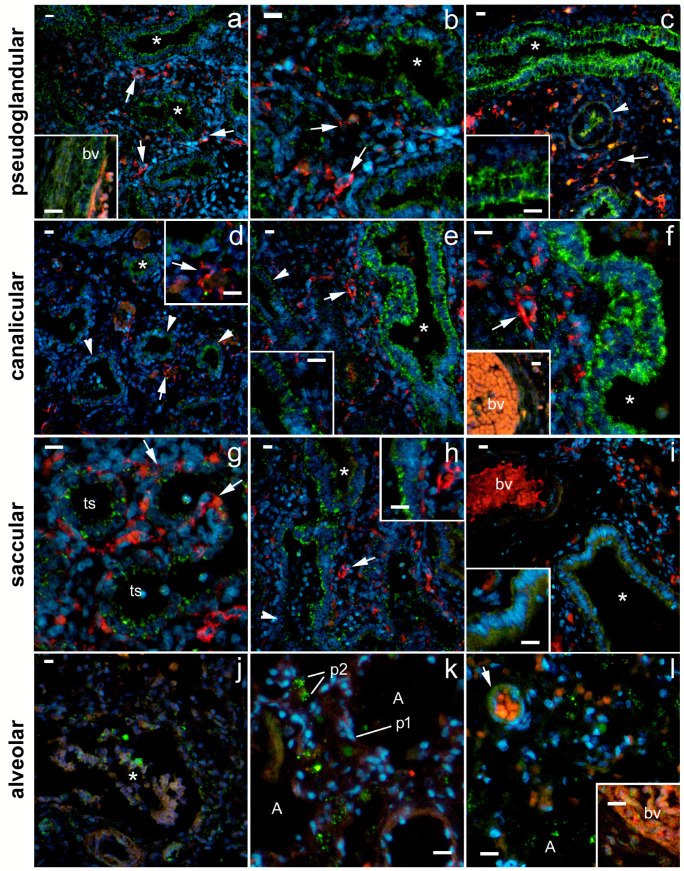
Immunofluorescence staining of fetal human lungs at different developmental stages: pseudoglandular (**a**–**c**), canalicular (**d**–**f**), saccular (**g**–**i**), and alveolar (**j**–**l**) with 5-HT3A and CD31/PECAM markers. Nuclear staining DNA DAPI is shown in parallel with 5-HT1A and CD31/PECAM immunofluorescence (merge). Green staining indicates positive expression of the 5-HT3A receptor, whereas red staining indicates expression of CD31/PECAM-1, and blue staining indicates nuclear staining with 6-diamidino-2-phenylindole dihydrochloride (DAPI). Images were taken at 400× magnification; (**a**,**c**) (main image), (**d**) (main image), (**e**) (main image), (**h**) (main image), (**i**) (main image), and (**j**) (downsized image). Scale bar = 20 µm. Asterisks—proximal airways/bronchioles; arrows—blood capillaries; arrowheads—distal airways, cuboidal epithelium lining the air spaces; bv—blood vessel (mostly artery); smc—smooth muscle cells; ts—end sacs; p1—pneumocyte type I; p2—pneumocyte type II; A—alveolus.

**Figure 7:**
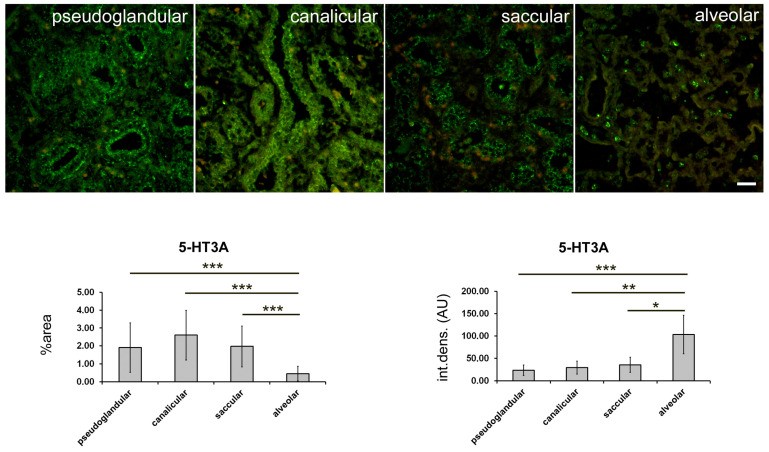
Quantification of 5-HT3A expression at different stages of fetal lung development. Immunofluorescence staining with 5-HT1A antibodies was performed. Representative images for different stages of lung development are shown. Green fluorescent staining—positive 5-HT3A receptor expression. Images were acquired at 400× magnification. Scale bar = 50 µm. Percentage of a sectional area with positive signal (% area) and integrated density (int. dens.; arbitrary fluorescence units—AU) were measured and are shown in graphs. * *p* < 0.05; ** *p* < 0.01; *** *p* < 0.001 between the indicated developmental stages (bars).

**Figure 8:**
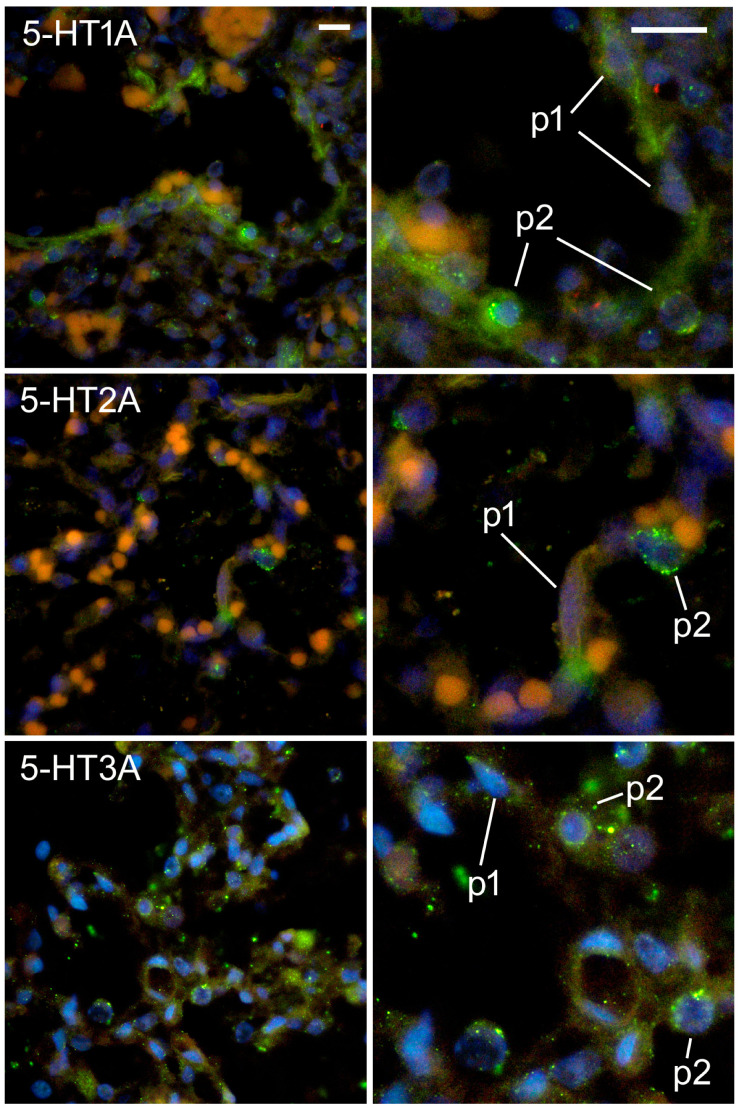
Immunofluorescence staining of fetal human lungs at the alveolar developmental stage with 5-HT1A, 5-HT2A, and 5-HT3A and CD31/PECAM markers. Nuclear DNA DAPI staining was combined with 5-HT1A. Green staining indicates positive 5-HT1A receptor expression, whereas blue staining indicates nuclear staining with 6-diamidino-2-phenylindole dihydrochloride (DAPI). Images were taken at 400× (**left** column) and 1000× magnification (**right** column). Scale bar = 20 µm, see all images in the same column. p1—type I pneumocytes; p2—type II pneumocytes.

The strongest expression for 5-HT3A was found in the epithelium of the proximal airways; it was equally strong in the pseudoglandular, canalicular, and saccular stages, and, unlike 5-HT1A and 5-HT2A, it was also maintained to a moderate extent in the bronchioles at the alveolar stage (Figure 6; Table 1). Moderate 5-HT3A expression was also observed in the arteries (endothelial and SMC) at the pseudoglandular and alveolar stages and in the SMC of the proximal airways at the alveolar stage. In addition, moderate 5-HT3A expression was found in the developing distal airways at the canalicular stage and strong 5-HT3A expression was found in the terminal saccule, which continued with a strong expression in the alveoli at the alveolar stage (Figure 6; Table 1). A statistically significant decrease in the percent area under the signal was observed at the alveolar stage, where it was lower compared with all three previous stages (*p* < 0.0001 for all; Figure 7). However, the integrated density (as a measure of the intensity of fluorescence) was significantly higher in the alveolar than in the pseudoglandular, canalicular, and saccular stages (*p* < 0.001 to *p* < 0.05). Careful observation revealed that the expression of 5-HT3A (similar to that of the other two receptors studied) had almost disappeared at the alveolar stage, but its strong expression was maintained in the pneumocytes of the alveolar wall, and was particularly strong in the pneumocytes of type II, although it was also present in the pneumocytes of type I (Figure 6 and Figure 8).

### 2.5. Expression of Serotonin Receptors in Pneumocytes

At the alveolar stage, a strong expression of 5-HT1A was observed in both type I and type II pneumocytes, although it was much stronger in type II pneumocytes (Figure 8). On the other hand, the expression of 5-HT2A was almost exclusively localized in type II pneumocytes, whereas it was absent or weak in type I pneumocytes (Figure 8). As mentioned earlier, a strong expression of 5-HT3A was found in both type I and type II pneumocytes, but it was much stronger in type II (Figure 8).

## 3. Discussion

Serotonin and its receptors have been found to be involved in the development of the lung and a variety of lung diseases. Disease-related alterations of the 5 HT system (in conjunction with genetic and/or environmental factors) have been found to lead to severe respiratory disorders at various stages of life, ranging from the early postnatal period with sudden infant death syndrome (SIDS) to neurodevelopmental disorders and to neurodegenerative diseases in the elderly and during old age [15]. A study in a mouse model of bleomycin-induced fibrosis showed the involvement of serotonin and its receptors in pulmonary fibrosis, with increased levels of serotonin being found in the lung and increased levels of serotonin receptors 5-HT2A and 5-HT2B being found in the fibrotic lungs [16]. Moreover, disruption of the pulmonary serotonin transporter has been associated with the pathogenesis of pulmonary hypertension in patients with chronic obstructive pulmonary disease [17].

Since the effect of serotonin depends on the type of receptor it activates [15,22], to understand the relationship between 5-HT and respiratory disorders, it is necessary to study the expression profile of the receptors of 5-HT. It has been found that 5-HT-im-munoreactive cells are present in human lungs from the eighth week of gestation [10]. However, little is known about the role of serotonin in fetal lung development and the expression and dynamics of serotonin receptors in the fetal lung during development. Therefore, we aimed to investigate the expression of three types of serotonin receptors (5-HT1A, 5-HT2A, and 5-HT3A) in the human fetal lung at different developmental stages.

The development of the vascular network is one of the most important processes for future lung function. The lung contains numerous receptors that play a role in the pathogenesis of pulmonary vascular remodeling [11]. The structure and function of the pulmonary vasculature change dramatically during fetal life. In the early pseudoglandular and canalicular stages of lung development, an adequate vascular network is required to allow adequate cell proliferation and to prevent tissue hypoxia. In contrast, a wide capillary network is required in the later stages to prepare for the formation of the blood–air barrier, which will be functional after birth. Vessel formation in the infant stage occurs in parallel with an increase in the surface area for gas exchange [23]. In addition, maturation of the capillary network and differentiation and maturation of the alveolar epithelium occur in the alveolar stage [23] alongside a decrease in proliferation and apoptosis [24,25,26]. The results of our study showed a strong expression of 5-HT1A in the endothelium of developing blood vessels in addition to its strong expression in the smooth muscle cells of the arterial tunica media. We can hypothesize that the previously described mitogenic effects of 5-HT1A (HT), which have been associated with intracellular reactive oxygen species (ROS) and/or MAPK-, Akt-, and Rho-associated protein kinase pathways (ROCK), may be one of the signals regulating endothelial proliferation during vascular network development. [27]. These findings were also consistent with a role for serotonin receptors in modulating vasomotility [11]. Indeed, serotonin is a potent direct constrictor of most vascular smooth muscle (primarily through the activation of 5-HT2A serotonin receptors), but it also increases endothelial cell production NO, which has vasodilatory effects [28]. Vasodilatory responses to serotonin have been observed in the arteries of different organs with different diameters [29]. A moderate expression of 5-HT1A and 5-HT2A was observed in the capillary endothelium at the pseudoglandular stage, whereas a strong expression of 5-HTA1 was observed in the endothelium of arteries at the pseudoglandular and alveolar stages and of 5-HT2A at the pseudoglandular and canalicular stages. The lowest expression in the endothelium was found for 5-HT3A, which was moderate in the pseudoglandular stage and low in the other stages. This may indicate that 5-HT1A and 5-HT2A play a dominant role in mediating serotonin effects on pulmonary vascular development. A similar pattern of expression was also observed in the arterial smooth muscle cells, with the exception of the pseudoglandular phase, when the smooth muscle cells showed a strong expression of the 5-HT1A receptor. The lower expression of 5-HT3A receptors in the vascular smooth muscle cells was consistent with the fact that 5-HT3 receptors are predominantly found in the presynaptic membrane of the neuromuscular junction [22]. Overall, these results supported a role for serotonin in blood vessel formation and the formation of the blood–air barrier during lung development.

During the pseudoglandular stage, the formation of the first 20 generations of the future lower airways and the onset of the acinar airways occurs [1,30,31]. During the canalicular stage, the most distal airways are formed and branching morphogenesis ends, which is followed by a decline in the surrounding mesenchyme. During this stage, surfactant production also begins in the type II alveolar cells. In the saccular stage, the epithelial surfaces for gas exchange increase greatly, and there is the formation of end-sacculi and a further differentiation of type I and type II alveolar cells with a maturation of surfactant production [1,32,33]. During the canalicular and saccular stages, apoptosis and proliferation peaks were detected in mesenchymal and epithelial cells, with mesenchymal apoptosis being accentuated in the canalicular stage [26]. The alveolar stage is characterised by an expansion of distal air spaces, repeated air space septation, and the formation of alveoli [34]. In the alveolar stage, there is a differentiation and maturation of the alveolar epithelium [23] and a decrease in proliferation and apoptosis [24,25,26].

In our study, we found a very strong expression of serotonin receptors in the epithelium of the proximal airways during the pseudoglandular, canalicular, and saccular phases and almost a complete extinction of 5-HT1A and 5-HT2A expression in the bronchial epithelium during the alveolar phase. The strongest 5-HT3A expression was found in the epithelium of the proximal airways. 5-HT3A expression was equally strong in the pseudoglandular, canalicular, and saccular stages. In addition, 5-HT3A expression was also present to a moderate extent in the bronchioles of the alveolar stage. The strong expression of serotonin receptors in the airways at the earlier stages of lung development may also be related to the aforementioned mitogenic effects of 5-HT3A [27] and may play a role in the branching and proximal and distal expansion of the airways.

A very strong expression of 5-HT1A was found in the respiratory SMCs at all the developmental stages. It is known that the effect of serotonin on the bronchial diameter depends on the type of receptors. In the control of human airway motility, 5-HT1A, 5-HT2A, 5-HT3, and 5-HT7 play important roles. 5-HT1A and 5-HT2A are mainly located in airway SMCs, while 5-HT3 and 5-HT7 are localized in presynaptic nerve terminals and facilitate the release of acetylcholine, which then acts on muscarinic receptors in SMCs [22]. In humans, activation of the 5-HT2A receptor on smooth muscle causes bronchoconstriction, whereas activation of the 5-HT1A receptor leads to bronchodilation [22,35]. A higher expression of these receptors at the earlier stages of lung development may be related to fetal respiratory movements that begin as early as day 10 after conception and are presumably critical for the mechanical stimulation required for lung development, particularly at the pseudoglandular stage [36].

The results of our study showed that all three of the types of serotonin receptors studied were strongly expressed by pneumocytes during the alveolar phase. The alveolar surface of the lung is lined with type I and type II alveolar epithelial cells. Type I alveolar epithelial cells (type I pneumocytes) serve as a physical barrier and a pathway for gas exchange, while type II pneumocytes produce surfactants and act as progenitor cells to replace injured type I alveolar epithelial cells [37]. Type I pneumocytes arise from the terminal differentiation of type II pneumocytes [38]. Type II pneumocytes are ideal for modulating immune responses because they are located at the boundary between the alveolar airspace and the lung interstitium (e.g., by producing cytokines and chemokines) [39]. It has been previously shown that primary type II pneumocytes are important regulators of the lung immune function, as they express the costimulatory molecules required for T cell activation and release IL-6 and the CXC chemokine CXCL8/ IL-8 in response to inflammatory stimuli [40]. In our study, we found that both types of pneumocytes expressed the serotonin receptors 5-HT1A and 5-HT3A in the alveolar stage, but the expression was much stronger in the type II pneumocytes. The expression of 5-HT2A was mainly localized in the type II pneumocytes, whereas it was weak in the type I pneumocytes.

In the present study, we observed that the percentage area of tissue covered with 5-HT2A and 5-HT3A receptor signal decreased significantly in the alveolar phase compared with the earlier developmental phases. The percentage of the 5-HT1A area and the intensity of fluorescence tended to decrease in the alveolar phase, but these differences were not statistically significant. Whereas the intensity of 5-HT2A fluorescence decreased, the intensity of 5-HT3A fluorescence increased significantly in the alveolar phase. These data suggested that as lung development progressed, the number of different cell types expressing serotonin receptors and responding to serotonin decreased, but their expression was strongly maintained in the pneumocytes, and 5-HT3A expression in these particular cells increased significantly in the alveolar phase. Further studies are needed to determine the role of serotonin and its receptors in alveolar epithelial cell function and a possible role in surfactant production and secretion by type II pneumocytes.

The main limitation of our study was the relatively small sample size and its retrospective nature. However, studies of human fetal tissue are not common because of the exclusive nature of the material and its often poor preservation. For these reasons, the sample size we had in this study was comparable to or larger than the sample sizes found in similar studies [20,21]. Therefore, we believe that the results of the present study can contribute to our knowledge of the serotonin receptor system in human lung development. However, to determine the exact role of each serotonin receptor during lung development, prospective experimental studies are needed, which would include a large number of samples and knock-out mouse models with the specific deletion of certain receptors in different cell populations.

## 4. Materials and Methods

### 4.1. Collecting Tissue Samples and Defining Fetus Gestation

Twenty-five fetuses ranging in age from 12 to 40 weeks of gestation were used for this study. Depending on the developmental stage, fetal lungs were divided into four groups: 8 samples from the pseudoglandular stage (12th–16th week of development), 7 samples from the canalicular stage (16th–26th week of development), 5 samples from the saccular stage (26th–36th week of development), and 5 samples from the alveolar stage (36th–40th week of development) [26,41]. The human material used came from the archival collection of the Department of Pathology, Cytology, and Forensic Medicine of the University Hospital in Mostar. Human fetuses were collected from the same department after spontaneous abortions. Only well-preserved and structurally normal fetuses without abnormal morphological signs or macerations were used for the study. After macroscopic examination, every 10th tissue section was stained with hematoxylin and eosin for additional control of tissue condition. The age of the fetuses was determined by foot length according to Streeter [42]. The study was performed in accordance with the Declaration of Helsinki and approved by the Ethical Committee of the School of Medicine University of Mostar (protocol code 01-1317/14, date 12 December 2014).

### 4.2. Sample Preparation

After fixation in 4% paraformaldehyde, the tissue was washed in phosphate-buffered saline (PBS) and then dehydrated in ethanol–water solutions with ascending concentrations. After clarification in xylene, the tissue was embedded in paraffin blocks, cut into 5 μm thick cross sections, and mounted on microscopic slides.

### 4.3. Immunohistochemistry and Immunofluorescence Staining

Lung sections were deparaffinized in xylene and then rehydrated in graded ethanol solutions and distilled water. Antigen retrieval was performed in a water steamer for 20 min at 95 °C with sodium citrate buffer (pH 6), which was then gradually cooled to room temperature and rinsed with PBS. A protein-blocking buffer (ab64226, Abcam, Cambridge, UK) was applied for 30 min at room temperature to prevent non-specific binding of the secondary antibody. Samples were then incubated overnight with primary antibody diluted in PBS (Table 2). The next day, the samples were washed in PBS and incubated for one hour at room temperature with appropriate secondary antibodies (Table 2). Finally, sections were stained with 4′,6-diamidino-2-phenylindole (DAPI) for nuclei detection before coverslipping (Immu-Mount, Thermo Shandon, Pittsburgh, PA, USA). When the primary antibodies were omitted from immunofluorescence, no staining was observed.

### 4.4. Data Acquisition and Statistical Analysis

An optical microscope (for H and E tissue sections) and a fluorescence microscope (Olympus BX51, Tokyo, Japan) with a Nikon DS -Ri1 camera (Nikon Corporation, Tokyo, Japan) were used to acquire images. Ten representative fields of view of the human fetal lung were acquired using the same camera settings and a 40× magnification. Images were processed using the ImageJ software (National Institutes of Health, Bethesda, MD, USA) and Adobe Photoshop (Adobe, San Jose, CA, USA). For the purpose of presentation, a slight subtraction of background and contrasting was performed.

Green staining indicated positive expression of 5-HT1A, 5-HT2A, and 5-HT3A receptors, whereas red staining indicated expression of CD31/PECAM-1. The intensity of staining in the different lung structures was assessed semiquantitatively using the following scale: no reactivity (−), mild reactivity (+), moderate reactivity (++), strong reactivity (+++), and very strong reactivity (++++) (Table 1). Proximal and distal airways (at the pseudoglandular, canalicular, and saccular stages) were distinguished on the basis of the epithelium: proximal airways had a columnar epithelium, and distal airways had a cuboidal epithelium.

Type I and type II pneumocytes were distinguished on the basis of their localization and morphological features. Both p1 and p2 were located in the alveolar wall (not in the lumen, unlike alveolar macrophages, or in the wall of vessels, unlike endothelial cells). p1 cells were flattened or squamous, whereas p2 cells were roundish and smaller than p1 cells (Figure 8) [38].

Photomicrographs were prepared for quantification of expression using a median filter (10 px) and a default thresholding algorithm after the initial subtraction of the red counter signal from the green fluorescence. The section percentage of area covered by the positive signal and the integrated signal density were measured using the ImageJ software. The PAST 4.03 software (Øyvind Hammer, Natural History Museum, University of Oslo, Norway) was used for statistical analyses, and a probability level of *p* < 0.05 was considered statistically significant. To compare the different developmental stages, a one-way ANOVA test followed by Tukey’s post hoc test was carried out for the comparison of the different developmental stages.

## 5. Conclusions

To the authors’ knowledge, this was the first study to address the spatial and temporal expression of the serotonin receptors 5-HT1A, 5-HT2A, and 5-HT3A in the human fetal lung. The dynamics of expression of the different types of serotonin receptors may be responsible for the fine regulation of serotonin actions during lung development, and their disruption could lead to various pathologies. The results presented provided us with new data on the development and function of the serotonin system in human lung development and gave us insight into its possible involvement in the pathogenesis of lung pathology, especially that characteristic of the neonatal period. The identification of defects in the serotonin signaling pathway could lead to new therapeutic strategies targeting their functions in infants with pulmonary hypertension. In this sense, knowledge of the dynamics of expression of different types of serotonin receptors during normal fetal lung development is a prerequisite for identifying abnormalities that may impair development and lead to respiratory disorders in early postnatal life.

## Figures and Tables

**Table 1:** Semi-quantification of serotonin receptor expression in different lung structures during fetal development.

	Developmental Stage	Proximal Airways /Bronchioli * —Epithelium	Proximal Airways /Bronchioli * —SMC	Mesenchime	Capillary Endothelium	Artery —Endothelium	Artery SMC	Distal Airways **	Alveoli ***	
									P1	P2
5-HT1A	Pseudoglandular	+++	++++	+	++	+++	++++	/	/	/
	Canalicular	++(+)	++++	−	+	−	+	++	/	/
	Saccular	+++	+++(+)	−/+	(+)	+	++++	+	/	/
	Alveolar	−	++(+)	/	+(+)	++++	+++	/	+(+)	+++
5-HT2A	Pseudoglandular	++	++	+	++	++(+)	++(+)	/	/	/
	Canalicular	+++	+	+	+	+++	+++	+	/	/
	Saccular	++	−/+	−/+	−/+	−/+	+(+)	+(+)	/	/
	Alveolar	−/+	−	/	−	−	−	/	−	+
5-HT3A	Pseudoglandular	+++(+)	−	+	+	++	++	/	/	/
	Canalicular	+++(+)	+	+	+	+	+	++	/	/
	Saccular	+++(+)	+	−/+	−	−	−	+++	/	/
	Alveolar	++	++	/	−/+	+(+)	++	/	−/+	++++

* Proximal airways at pseudoglandular, canalicular, and saccular stages, bronchioles at alveolar stage; SMC—smooth muscle cells; ** only at pseudoglandular, canalicular, and saccular stages; *** alveolar stage; P1—type I pneumocytes; P2—type II pneumocytes. The intensity of staining in different lung structures was evaluated semiquantitatively according to the following scale: no reactivity (−), mild reactivity (+), moderate reactivity (++), strong reactivity (+++) and very strong reactivity (++++).

**Table 2:** Primary and secondary antibodies used in the study.

	Antibody	Code No.	Host	Dilution	Source
Primary	Anti-5HT1A receptor antibody	ab227165	Rabbit	1:150	Abcam, Cambridge, UK
	Anti-SR-2A (A-4)	sc-166775	Mouse	1:150	Santa Cruz Biotechnology Inc., Santa Cruz, CA, USA
	Anti-5HT3A receptor antibody	GTX54151	Rabbit	1:150	GeneTex, Irvine, CA, USA
	Anti-CD31/PECAM-1 (H3), Alexa fluor 546 conjugated	sc-376764	Mouse	1:50	Santa Cruz Biotechnology Inc.
	Anti-CD31/PECAM-1	NB100-2284	Rabbit	1:100	Novus Biologicals Englewood, CO, USA
Secondary	Anti-Mouse lgG, Alexa Fluor^®^488	715-545-150	Donkey		Jackson Immuno Research Laboratories, Inc., Baltimore, PA, USA
	Anti-Rabbit lgG, Alexa Fluor^®^488	711-545-152	Donkey		Jackson Immuno Research Laboratories, Inc., Baltimore, PA, USA
	Anti-Rabbit IgG, Rhodamine Red™-X	711-295-152	Donkey		Jackson Immuno Research Laboratories, Inc., Baltimore, PA, USA

## Data Availability

Data are available from the corresponding author upon the reasonable request.
